# Challenges and future of HER2-positive gastric cancer therapy

**DOI:** 10.3389/fonc.2023.1080990

**Published:** 2023-01-30

**Authors:** Chenzhe Ma, Xiao Wang, Jiwu Guo, Bo Yang, Yumin Li

**Affiliations:** ^1^ The Second Clinical Medical College, Lanzhou University, Lanzhou, China; ^2^ Key Laboratory of the Digestive System Tumors of Gansu Province, Lanzhou University Second Hospital, Lanzhou, China

**Keywords:** gastric cancer, receptor ErbB-2, HER2, Her2 resistance, trastuzumab

## Abstract

Gastric cancer is the fifth most common cancer worldwide, and the treatment of advanced gastric cancer has relatively little progress. With the continuous development of molecularly targeted therapy for tumors, it has been discovered that human epidermal growth factor receptor 2 (HER2) contributes to the poor prognosis and pathogenesis of various cancers. In order to treat HER2-positive advanced gastric cancer, Trastuzumab has emerged as the first first-line targeted medication used in conjunction with chemotherapy. The consequent trastuzumab resistance has become an important issue, and various new HER2-targeted gastric cancer drugs are emerging to address this challenge. This review’s primary concern is the drug mechanism of various HER2-positive gastric cancer targeted therapy and fresh techniques of detection.

## 1 Introduction

Since 2010, China’s cancer incidence and fatality rates have increased, making cancer the leading cause of death and a major public health issue (1). Gastric cancer is the second most common cancer causing death in China, and its 5-year survival rate is very low because more than 80% of patients are diagnosed with advanced gastric cancer (AGC) (2). The 5-year survival rate for AGC is less than 10%, and even if novel chemotherapy protocols and biological therapy are being used, median overall survival (OS) is still less than 1 year (3). Gastric cancer remains important worldwide, causing over 1 million new cases and an estimated 769,000 deaths in 2020, incidence and mortality rank fifth and fourth in the world, respectively (4). Only in recent years has it been discovered that *H. pylori* infection can lead to antral/body gastric cancer. Additionally, *H. pylori* infection greatly increases the risk of developing gastric cancer. The risk of gastric cancer brought on by a chronic *H. pylori* infection may also be influenced by additional risk factors, such as smoking, excessive salt consumption, and drinking alcohol (5, 6).

Different perioperative treatment strategies (neo-adjuvant, adjuvant, or both) have improved survival for patients with locally AGC. For unresectable gastric cancer, In first-line therapy, the combination of platinum compounds with fluoropyrimidine-based chemotherapy is successful in extending survival, improving symptoms, and enhancing the quality of life (7). Adding a third drug, such as docetaxel, to the platinum-fluoropyrimidine combination increases toxicity but improves patient survival (8). With the application of various novel immunotherapy and molecular targeted therapy, anti-HER2 is the most widely used target because it significantly increases the survival of cancer patients.

## 2 The HER2 pathway and the relevance of gastric cancer

**Figure 1:**
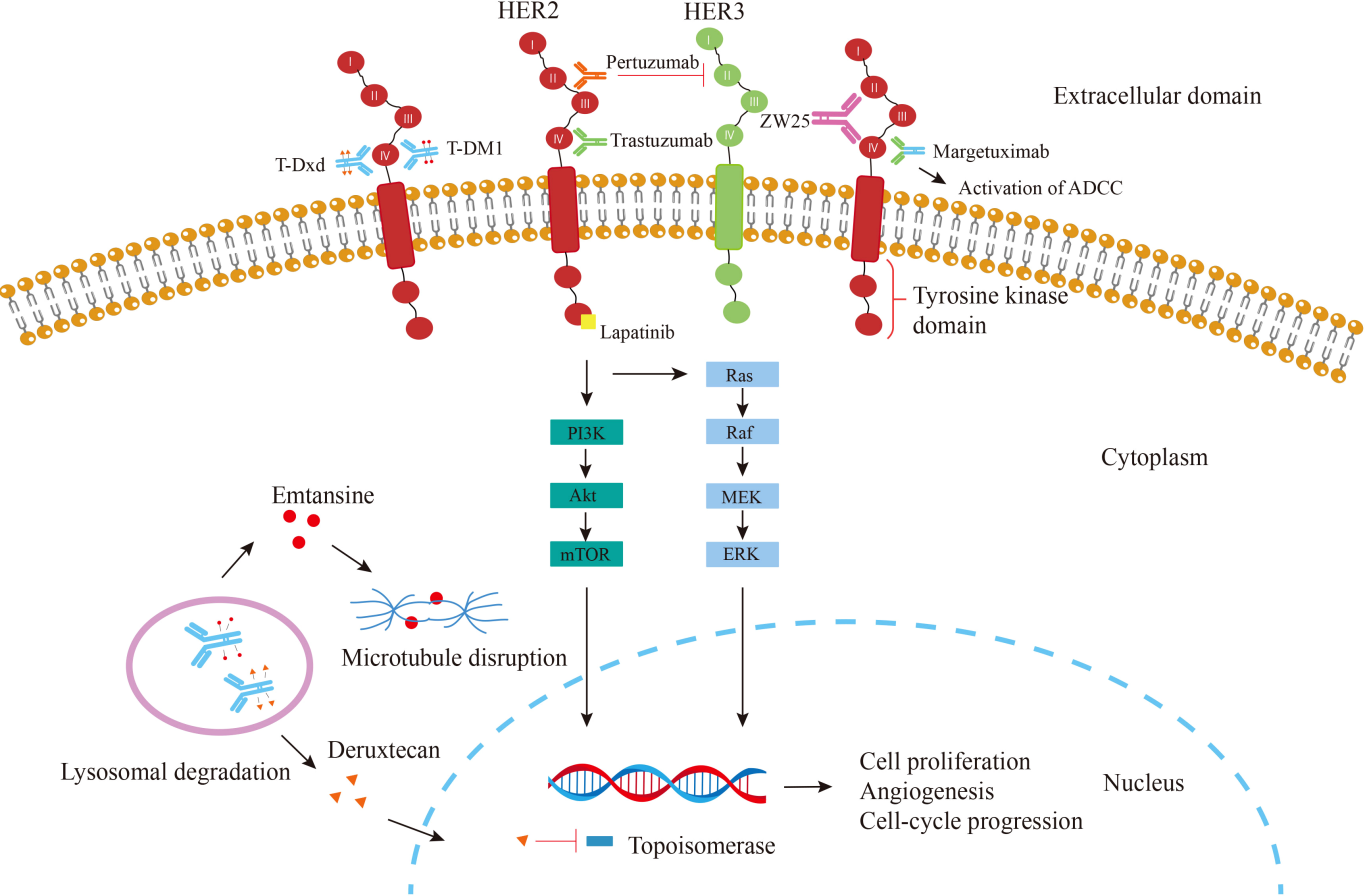
Mechanism of action of agents targeting HER2. Trastuzumab (green) binds to the ECD IV of HER2, thereby inhibiting the HER2 signaling pathway leading to cell cycle arrest; Pertuzumab (orange), which binds to the ECD II and Inhibits dimer formation; Margetuximab, which binds trastuzumab to an altered Fc-γ domain that involved in ADCC; Lapatinib (yellow) directly prevents the PI3K pathway from being activated by binding to the intracellular tyrosine kinase domain of HER2; T-DM1 (red circle)releases the emtansine moiety after the ADC is phagocytosed by lysosomes; T-Dxd (orange triangle), another ADC combining trastuzumab and deruxtecan, a potent topoisomerase I inhibitor; Bispecific antibody ZW25 (purple) binds to both extracellular domains II and IV of HER-2.

**Figure 2:**
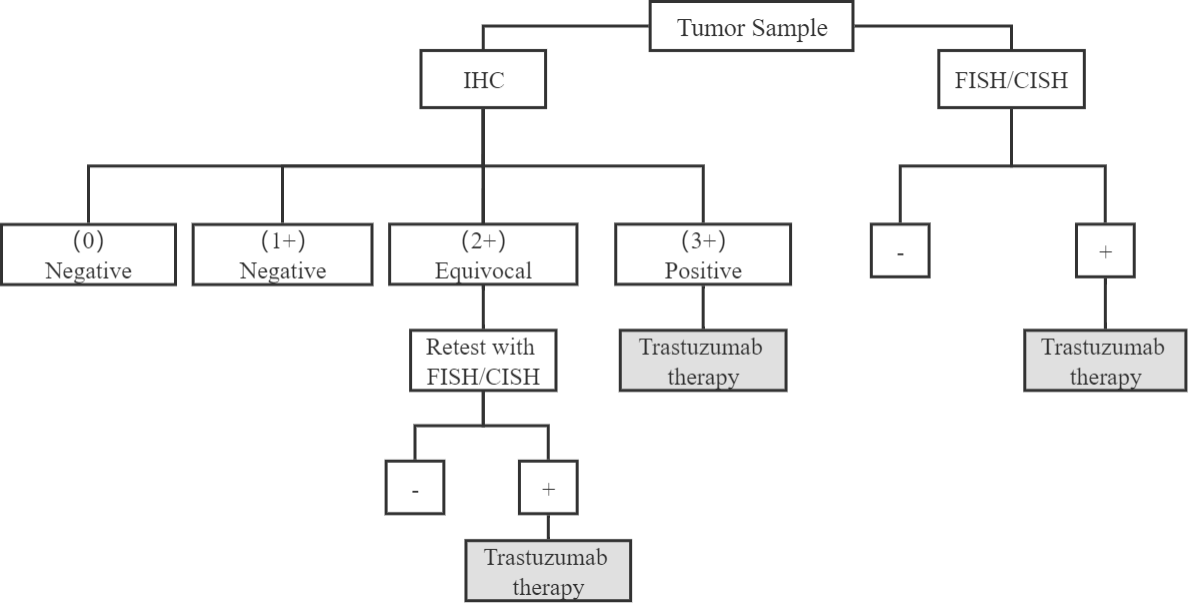
HER2/neu testing algorithm.

Human epidermal growth factor receptor 2 (HER2), also known as Neu or ErbB2, Encoded by ERBB2 on chromosome 17, is a transmembrane tyrosine kinase (TK) receptor belonging to the epidermal growth factor receptor (EGFR) family, This family consists of four members (HER1 or EGFR, HER2, HER3, and HER4), all of which have an extracellular domain(ECD), a transmembrane domain, and an intracellular kinase domain (9, 10). (**Figure 1**.) The binding of different ligands to the ECD initiates a series of signal transduction pathways, which are crucial for the growth, apoptosis, adhesion, migration, and differentiation of tumor cells (11). The HER2 receptor, first discovered in 1984, is a 185kD transmembrane glycoprotein (12). HER2 lacks ligand-binding activity and requires heterodimerization with other family members (HER1 and/or HER3) to be activated (13). Among them, the HER2-HER3 heterodimer is the most active HER signaling dimer and plays a crucial role in HER2-driven tumor oncogenic transformation, HER-2 activates downstream pathways through heterodimerization and tyrosine kinase autophosphorylation mediated signal transduction, major signaling pathways include Ras/MAPK and PI3K/Akt (14). They are essential pathways regulating cell proliferation, differentiation and survival and are closely related to the pathogenesis of various tumors (15–17). HER2-specific antibodies interfere with HER2 signaling primarily by blocking heterodimerization between ErbB-2 and growth factor receptors (18). Use immunohistochemistry (IHC) to detect HER2 protein, fluorescence *in situ* hybridization (FISH) or chromogenic *in situ* hybridization (CISH) to detect gene amplification, HER2 overexpression can be classified as IHC0 (negative), IHC1+ (negative), IHC2+ (equivocal), or IHC3+ (positive), and samples with an IHC 2+ value should undergo another FISH or CISH test(**Figure 2**) **(** 19, 20).

Although non-small cell lung cancer (NSCLC), ovarian, colon, and pancreatic cancers overexpress HER2 protein and/or show gene amplification in varying proportions of cases, HER2 protein overexpression is most pronounced in breast and gastric cancers (21). In comparison to HER2-negative breast cancer, HER2-positive breast cancer is more well-studied and has greater mortality and risk of distant metastases. Following its approval in 1998, trastuzumab, one of the first molecularly targeted drugs to be developed, is now recommended for all patients with early-stage HER2-positive disease and has a better prognosis (22–24). According to the latest global report, the average HER2-positive rate of gastric cancer is 17.9%, of which the HER2-positive rate in Chinese gastric cancer patients was 8.8% (25, 26). HER2 overexpression was associated with tumor location, tumor differentiation, Bormann classification, Lauren’s classification, lymph node status, venous invasion, and lymphatic invasion in patients. Among gastric cancers with high expression of HER2 protein, gastroesophageal junction cancer(GEJ) is more common than gastric corpus cancer. Predominantly tubular adenocarcinoma (larger proportion of well/moderately differentiated carcinomas, lesser proportion of poorly differentiated adenocarcinomas), and HER2 positivity is more common in the Intestinal type than diffuse or mixed type. However, there is no correlation with gender, age, or clinical stage (27–30). However, there is controversy over the role of HER2 in predicting prognosis in gastric cancer, as opposed to the poor prognosis associated with HER2 positivity in breast cancer. Studies have revealed that the prognosis is independent of HER2 expression. Therefore, HER2 remains an uncertain predictor of gastric cancer prognosis, and more research is required (31–33).

## 3 Monoclonal antibodies

### 3.1 Trastuzumab

Patients with HER2-positive gastric cancer have seen favorable clinical results after receiving anti-HER2 therapy. Trastuzumab is a humanized recombinant monoclonal antibody that selectively binds to HER2 ECD IV and reduces the expression of HER2 receptors, Thereby inhibiting angiogenesis, reducing DNA repair, and inducing apoptosis. On the other hand, trastuzumab contains an IgG1 Fc structure, which Can mediate antibody-dependent cellular cytotoxicity (ADCC) to attack target cells (34). Patients who underwent chemotherapy with cisplatin and fluorouracil in combination with trastuzumab had a better median OS than those who just got chemotherapy(16 months *vs* 11 months). This is mainly due to the survival advantage of patients with high expression of the HER2 protein (35, 36).

With the advancement of tumor immunotherapy, combined immune checkpoint inhibitors will emerge as a promising treatment. A phase II trial (NCT03409848) revealed that trastuzumab and PD-1 inhibitors nivolumab combined with first-line chemotherapy showed promising efficacy in HER2-positive GEJ Cancer (37). Meanwhile, pembrolizumab can also be safely used in combination with trastuzumab and chemotherapy (38). Pembrolizumab was added to trastuzumab and chemotherapy in a phase III trial (NCT03615326), which dramatically decreased tumor size and improved objective response rates (39).

The issue has gained attention as most patients develop resistance to trastuzumab. Trastuzumab resistance appears to be primarily mediated by tumor heterogeneity. Treatment failure with anti-HER2 therapy is also associated with changes in receptor tyrosine kinase-RAS-PI3K signaling. Additionally, Mucins, which are cell surface proteins, reduce the HER2 receptor’s interaction with trastuzumab. As a result, the drug’s inhibitory effect is blocked (31, 40). To overcome this problem, a variety of new drugs and treatments are emerging.

### 3.2 Pertuzumab

Pertuzumab is a recombinant humanized anti-HER2 antibody with different antitumor activity than trastuzumab. Pertuzumab binds to the ECD II of HER2. Therefore, pertuzumab can inhibit the dimerization of HER2 with other HER family members, especially effectively block HER2-HER3 heterodimerization, thereby preventing ligand-dependent HER2 signaling (41–43). Adding pertuzumab to trastuzumab and chemotherapy has not been found to increase survival in patients with HER2-positive metastatic gastric cancer, despite the fact that pertuzumab significantly prolongs the lives of patients with metastatic breast cancer. This may be a consequence of the different tumor biology exhibited by HER2-positive AGC and HER2-positive breast cancer (44, 45). The ongoing INNOVATION trial will individually evaluate the relative benefit of trastuzumab and pertuzumab in perioperative therapy (45).

### 3.3 Margetuximab

Margetuximab is a HER2-targeted antibody with an engineered FCγ domain. Special modification of the Fc region increases its binding to the activating Fc receptor FcγRIIIA (CD16A) and reduces its binding to the inhibitory Fc receptor FcγRIIB (CD32B). Therefore, the response rate is increased (46). CD16A is an Fc receptor important in mediating ADCC effects, which renders margetuximab more potent and more cytotoxic than trastuzumab with the wild-type Fc domain. A phase Ib/II trial(NCT02689284) has shown that margetuximab combined with anti-PD-1 drugs such as pembrolizumab and retifanlimab have synergistic antitumor activity (47). At the same time, it has a stronger impact on people with tumors expressing low HER2 or those with CD16A low binding alleles (48). In addition, compared with trastuzumab, margetuximab can enhance the ADCC effect to produce a stronger killing effect on tumor cells (49). The current Phase II/III, randomized trial(MAHOGANY) is assessing margetuximab plus retifanlimab with/without chemotherapy and margetuximab plus tebotelimab with chemotherapy in HER2-Positive Gastric or GEJ Cancer (50).

## 4 TKIs

Tyrosine kinase inhibitors (TKIs) compete with ATP for the ATP binding site of Protein tyrosine kinase(PTK), leading to a reduction in tyrosine kinase phosphorylation and blocking downstream signaling pathways, thereby inhibiting cancer cell proliferation (51, 52). Lapatinib, a small-molecule inhibitor of the EGFR and HER2 tyrosine kinase domains, was first approved for treating HER2-positive breast cancer (53). In treating HER2-positive gastric cancer, the median OS of capecitabine and oxaliplatin (CapeOx) combined with lapatinib and CapeOx plus placebo was 12.2 months and 10.5 months, respectively, and there was no significant difference. Median progression-free survival (PFS) was 6.0 months and 5.4 months, respectively (54). On the other hand, the median OS of lapatinib plus paclitaxel and paclitaxel alone was 11.0 months and 8.9 months, respectively (P=0.1044). There were also no significant differences in PFS (5.4 months *vs*. 4.4 months) and time to progression (TTP) (5.5 months *vs*. 4.4 months). Therefore, the addition of lapatinib to CapeOx’s first-line chemotherapy regimen and second-line regimens of lapatinib plus paclitaxel did not improve patients’ OS (55). Afatinib irreversibly inhibits the ErbB family, paclitaxel and afatinib are being tested in a trial (NCT01522768) for patients with HER2-positive, trastuzumab-refractory esophagogastric cancer.

## 5 Antibody-drug conjugates

### 5.1 Trastuzumab emtansine

Antibody-drug conjugates (ADC) is an emerging antibody bioconjugate, which is an immunoconjugate composed of a monoclonal antibody bound to a cytotoxic drug through a chemical linker, combining the antigen specificity of the antibody and the potency of the cytotoxic agent at the same time (56). Monoclonal antibodies are used as carriers to target cytotoxic drugs to specific cells. The antibodies bind to specific receptors on the surface of the target cells and are then degraded by lysosomes after endocytosis. Intracellular small-molecule cytotoxic drugs are released in large quantities, destroying DNA chains or microtubules, or exerting topoisomerase or RNA polymerase inhibitory effects, resulting in tumor cell death (57, 58). Trastuzumab emtansine (T-DM1) is a kind of ADC composed of trastuzumab linked to the tubulin inhibitor DM1 (a derivative of maytansine) through a stable linker. Catabolites containing cytotoxic emtansine are released intracellularly to induce mitotic arrest and apoptosis. However, AGC patients treated with T-DM1 did not have a clear advantage in OS compared with patients treated with taxanes (59, 60).

### 5.2 Trastuzumab deruxtecan

Trastuzumab deruxtecan (T-Dxd) is a novel ADC consisting of a humanized anti-HER2 antibody covalently linked to a topoisomerase I inhibitor (DXd) *via* a tetrapeptide-based cleavable linker (61). The ADC payload (ie, DXd) was delivered directly to HER2-expressing tumor cells, reducing damage to normal cells by cytotoxic agents (62). Unlike T-DM1, T-Dxd showed stronger antitumor activity against gastric or GEJ cancer with low HER2 expression (63). Compared with conventional chemotherapy, T-Dxd significantly improved patient response rate (RR) (51% *vs* 14%) and prolonged OS(12.5 months *vs* 8.4 months) (64). Other studies have shown that T-Dxd is not only effective against HER2 protein-positive tumor cells but also effective against HER2-negative tumor cells in the presence of HER2-positive cells. Due to the high membrane permeability of T-Dxd, this bystander-killing effect may be due to T-Dxd being internalized by HER2-positive cells, and DXd being released into the cytoplasm and then being transferred to adjacent HER2-negative cells (65, 66). Ongoing Phase II trials (NCT04014075) and (NCT04379596) will study safety and efficacy of T-Dxd drug alone or in combination with chemotherapy and/or immunotherapy.

## 6 Emerging treatments

### 6.1 ZW25

ZW25 is a bispecific antibody that simultaneously binds two HER2 epitopes: ECD4 and ECD2. Compared with trastuzumab or pertuzumab, ZW25 has stronger antitumor activity, can effectively silence HER2 signaling, and also stimulate the immune system. ZW25 has been demonstrated to have single-agent action and to be well tolerated (67). A clinical trial recruiting 24 patients with HER2-positive cancer, 71% of whom had previously received trastuzumab, showed a median PFS of 6.2 months and a disease control rate of 82%. Diarrhea, infusion reactions, and nausea were the most common grade 1 or 2 side effects. Phase II trials will test the medication both by itself and when combined with chemotherapy (68). Additionally, a trial combining ZW25 with tislelizumab and chemotherapy is ongoing (NCT04276493).

### 6.2 CAR-T therapy

Chimeric antigen receptor T (CAR-T) cell therapy is an adaptive cellular immunotherapy in which CAR-redirected T cells expressing engineered receptors for specific antigens are reinfused into patients, thereby triggering an effective antitumor immune response (69). Major histocompatibility complex (MHC)-independent tumor-associated antigen recognition is made feasible by CARs (70). CARs consist of an extracellular target antigen-binding domain, a hinge region transmembrane domain, and one or more intracellular signaling domains (71). Among them, chimeric antigen receptor-modified T cell therapy targeting CD19 is very effective in relapsed acute lymphoblastic leukemia (72). Due to the substantial heterogeneity of gastric cancer cells, there is not much research on the application of CAR-T cells in the treatment of gastric cancer. A study showed that expanded CAR-T cells efficiently eliminated HER2-positive gastric cancer cells from patients after being specifically triggered by the HER2 antigen (73). In HER2-positive xenograft tumors, CAR-T cells’ tumor suppressor and killing abilities were significantly enhanced compared with non-transduced T cells (74). HER2-targeted CAR-T cells for treating HER2-positive AGC is a promising therapeutic strategy, but additional study is required to determine its toxicity and immunogenicity. Patients with refractory HER2-positive solid tumors will participate in a phase I study(NCT04511871) to investigate the CAR-T’s safety and preliminary therapeutic efficacy.

## 7 Drug resistance in anti−HER2 therapy

### 7.1 HER2 heterogenous expression

Intratumor heterogeneity and genomic instability processes shape tumor evolution in space and time, and growing evidence suggests a link between assessment heterogeneity and poor prognosis. This explains the mismatch between the costs and benefits of some cancer treatments (75). Gastric cancer tumor cells have greater HER-2 heterogeneity (from 26 to 79% in IHC) compared to breast cancer (76). HER-2 heterogeneity may reduce the efficacy of trastuzumab. Two Japanese studies discovered it to be an independent predictor of poor prognosis (77). This may be the most important primary mechanism of anti-HER2 drug resistance.

### 7.2 Loss of HER2 positivity

HER2 loss is one of the primary causes of acquired resistance to trastuzumab in HER2-positive gastric cancer patients (78). In patients with HER2-positive gastric cancer receiving trastuzumab, 29.1%-64% of patients developed loss of HER2 expression during treatment (IHC score <3+ and absence of ISH amplification) and/or loss of HER2 overexpression (IHC “down scoring” from 2+/3+ to 0/1+), At the same time, the heterogeneity of HER2 gene expression increased. This phenomenon was found more frequent in tumors with an initial IHC score of 2+, suggesting that HER2 status needs to be reassessed before starting second-line anti-HER2 therapy (79, 80).

### 7.3 Activation of alternative pathways

Src, a non-receptor tyrosine kinase of the Src family, plays a role in signaling and crosstalk between growth-promoting pathways (81). Studies have shown a relationship between the low clinical effect of trastuzumab and the changes of the PI3K/Akt pathway. Acquired resistance to trastuzumab in both HER2-overexpressing breast and gastric cancer are associated with sustained Src-mediated activation of the MAPK/ERK and PI3K/mTOR pathways (82, 83). Meanwhile, HER3 overexpression was observed in drug-resistant gastric cancer cell lines, which may be induced by HER2 blockade (84). Therefore, blocking these targets may improve drug resistance in patients.

### 7.4 Epithelial to mesenchymal transition

Epithelial to Mesenchymal Transition (EMT) is a reversible cellular program that temporarily places epithelial cells in a quasi-mesenchymal state (85). EMT is essential for the invasion and metastasis of cancer cells as well as embryogenesis and the healing of wounds (86, 87). EMT and HER4 may be associated with resistance to HER2 therapy. HER4 and phosphorylated HER4 (p-HER4) expression was increased in trastuzumab-resistant cells while activating the downstream PI3K pathway through the HER4-YAP1 axis to promote the transformation of epithelial cells to mesenchymal cells to maintain the invasiveness of HER2-positive gastric cancer and escape the blockade of trastuzumab (88, 89).

## 8 New screening techniques

### 8.1 Circulating tumor DNA

To better assess patient outcomes, we need improved diagnostic, prognostic, and disease surveillance methods despite the availability of various treatments (90). Circulating tumor DNA (ctDNA) is released into the bloodstream by circulating tumor cells during tumor cell apoptosis or necrosis, a process that occurs before the tumor is detected by imaging means or clinical symptoms are not manifested. As a result, ctDNA is one of the most promising biomarkers for detecting cancer in its early stages (91, 92). In a study of ctDNA in early gastric cancer, HER2 amplification in tumor tissue and DNA samples matched at a rate of roughly 60% (93). With the application of modern technologies such as digital droplet PCR and next generation sequencing, the coincidence rate between ctDNA and her2 expression in tumor tissue has increased to about 90% (90, 94). Moreover, the detection of ctDNA can overcome the heterogeneity of some tumors, which suggests that the targeted HER2 population can be screened alternatively using ctDNA (95). Based on the advantages of non-invasive and dynamic monitoring of ctDNA, it can be used as a tool to evaluate and predict the effectiveness of anti-HER2 therapy (96).

### 8.2 Circulating tumor cells

Circulating tumor cells (CTCs) are tumor cells that shed into the blood and circulate throughout the body (97). The spread and migration of CTCs are important causes of distant metastasis of tumors (98). CTCs counting is a non-invasive method to monitor chemotherapy response and real-time progression, which is more valuable in predicting the sensitivity or prognosis of AGC patients to chemotherapy drugs (99–101). Recent research has found that HER2 amplification detected in CTCs is highly consistent with patient tissue. Therefore CTCs can serve as a non-invasive alternative to document gene amplification in GC patients (102–104).

### 8.3 ^89^Zr-Trastuzumab PET/CT

^89^Zr-Trastuzumab PET/CT is an imaging test using radionuclide ^89^Zr-labeled trastuzumab as an imaging agent to determine HER2 expression heterogeneity in gastric cancers and the effect of HER2-targeted therapy. ^89^Zr-Trastuzumab has additional advantages over single-point biopsy, allowing simultaneous non-invasive assessment of changes in HER2 levels and target binding in the primary tumor and all metastatic sites (105–107). HER2 PET imaging will play an important role in improving diagnosis, staging (eg, when lesions cannot be biopsied), guiding individualized therapy, and the development of targeted drugs (108).

## 9 Conclusion and prospects

The role of HER-2 in gastric cancer has been demonstrated, and HER2-targeted therapy has dramatically improved the prognosis for patients with early- and late-stage HER2-positive AGC. But only trastuzumab prolongs OS and PFS and is approved as the first-line standard of care. Furthermore, gastric cancer’s intratumoral, intrapatient, and interpatient heterogeneity remains a crucial obstacle to developing targeted therapy drugs. At the same time, immune checkpoint inhibitor monotherapy is not effective against the majority of gastric cancer, hence a combination of immunotherapy and anti-HER2 monoclonal antibodies may be required. To overcome these challenges, novel HER2-targeted drugs have been developed, such as ADCs, TKIs, and bispecific antibodies. New screening methods, such as ctDNA and new imaging agents, allow real-time assessment and monitoring of anti-HER2 treatment in a less invasive manner. Overall, the outcomes for both present and future patients will be significantly improved by new research approaches that address the issues mentioned above.

## Author contributions

CM: Writing- Original draft preparation, Investigation, and figure preparation. XW: data collection. JG: Investigation. BY: data collection. YL: Conceptualization, Methodology, Supervision. All authors contributed to the article and approved the submitted version.
